# Open sternal fracture with bilateral dislocation of the sternoclavicular joints after a high-speed bicycle accident: a case report

**DOI:** 10.1186/s13256-024-04877-6

**Published:** 2024-11-10

**Authors:** Christian Prangenberg, Alberto Alfieri Zellner, Jonas Roos, Donatas Zalepugas, Robert Ossendorff, Soufian Ben Amar, Davide Cucchi, Sebastian Scheidt

**Affiliations:** 1 Clinic for Orthopedics and Trauma Surgery of the University Hospital Bonn, Venusberg Campus 1, 53127 Bonn, Germany; 2 Clinic for General, Visceral, Thoracic and Vascular Surgery of the University Hospital Bonn, Venusberg Campus 1, 53127 Bonn, Germany

**Keywords:** Trauma, Thoracic surgery, Traumatology, Road accident, Polytrauma, Sternoclavicular joint, Emergency medicine

## Abstract

**Background:**

Only a few cases of bilateral traumatic sternoclavicular dislocations have been reported in the literature. This injury is considered one of the rarest injuries of the human musculoskeletal system. Therefore, we present the first documented case of a cyclist with a third-degree open thoracic trauma (Gustilo–Anderson 3a) associated with a dislocated manubrium sterni fracture in the upper thoracic aperture, bilateral anterior dislocations of the sternoclavicular joints, rib fractures, and pleural ruptures.

**Case presentation:**

The patient, a 27-year-old Caucasian male, incurred this injury while participating in a professional cycling race at Nürburgring, Germany and received immediate interdisciplinary surgical treatment and has encountered no complications. We conducted a 1-year follow-up and present the clinical findings of this follow up. Additionally, we conducted a comprehensive review of the existing literature on this injury.

**Conclusions:**

Immediate interdisciplinary intervention, including surgical repair and meticulous postoperative care, facilitated successful patient recovery. This underscores the critical role of comprehensive trauma management in complex polytrauma cases. In conclusion, this case report highlights the rarity and complexity of a traumatic injury involving bilateral sternoclavicular dislocation, with this case being the first case reported with concomitant open thorax trauma. Our patient benefited greatly from immediate air-bound transportation to an interdisciplinary care provider, which houses both thoracic and trauma surgery departments.

## Background

Due to the growing ecological awareness among the population, there has been a shift towards nonmotorized transportation in European cities [1–3]. Consequently, the use of bicycles as means of transportation has increased, leading to a rise in bicycle accidents. The majority of these accidents occur near home or on the road [4], and often involve cyclists not wearing protective gear. The mortality rate on admission after a bicycle accident is 5.7% [2]. Over half of the patients sustain multiple injuries to their extremities. Clavicle fractures are a common occurrence, with 3% of such shoulder girdle injuries also involving a dislocation in the sternoclavicular joint [5]. Bilateral dislocations of the sternoclavicular joint have only been described nine times in the literature. The human thorax has a remarkable ability to withstand injury. In clinical practice, rib fractures are observed in a range of scenarios, from minor accidents in geriatric patients to work-related falls and moderate to high-speed road traffic accidents in younger patients. High-speed sports accidents are the most common cause of rib fractures, which are typically single fractures, but can also be serial rib fractures [4].

In this report we present an extremely rare case involving a third-degree open chest injury with ventral thoracic compression, dislocated fracture of the manubrium sterni AO 16.3.1A, dislocated fractures of the ventral ribs AO 16.1.2.3A, and bilateral anterior dislocation of the clavicles. In this case the horizontal fracture of the sternum and bilateral sternoclavicular dislocation required an interdisciplinary surgical approach. We believe that the patient benefitted greatly by the multidisciplinary approach adopted for timely and effective management. To our knowledge, the simultaneous presence of these injuries in a single patient has not yet been reported in the medical literature, making this case an interesting topic for colleagues. Interesting aspects of the surgical treatment in this case include off label use of implants for this rare kind of fracture as well as sternoclavicular joint (SC-joint) stabilization.

## Case presentation

### Accident and emergency room

This article reports the case of a 27-year-old Caucasian male patient who participated in the “Rad am Ring” bicycle race at the famous Nürburgring racetrack in Germany.

The patient was helicoptered into our level one trauma center as intubated, ventilated, and hemodynamically stable after sustaining open chest trauma following a bicycle accident at the Nürburgring racetrack. The emergency room staff, including trauma surgeons, visceral and thoracic surgeons, an anesthesiologist, and a radiologist, provided immediate interdisciplinary care. The trauma team and thoracic surgery department performed the operative treatment.

**Fig. 1:**
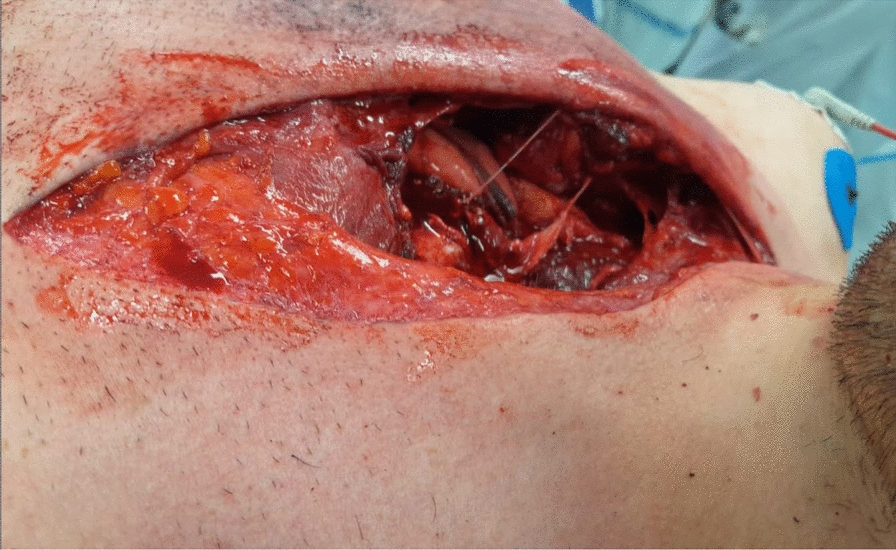
Open thorax trauma extending from the right clavicle to the sternum. Traumatic rupture of the right pectoralis major, manubrium sterni fracture with bilateral sternoclavicular (SC) joint dislocation. Traumatic rupture of the right ventral pleura parietalis with open pneumothorax of the right lung

The accident occurred on a downhill section of the racetrack on stormy weather. The patient, who was only wearing a helmet for protective gear, lost control of his bicycle traveling at around 74 km/h, managed to slow down to 55 km/hour and subsequently crashed hitting the guardrail of the track. On arrival at the scene, paramedics found the patient alert and responsive. The injury was a large laceration in the chest with an open chest cavity and a visible heartbeat. As the patient was becoming more disoriented by the minute and had a Glasgow coma scale of 11 upon arrival of the medical staff, the patient was anesthetized with ketamine (Ketanest^®^, Pfizer Pharma) and midazolam (Dormicum^®^, Roche Pharma) and the patient’s airway was secured by intubation, the cervical spine and full body were immobilized with a stiff neck and a vacuum mattress. Two peripheral intravenous lines were inserted to further stabilize the patient through fluid resuscitation. The thoracic incision was covered with a sterile compressive dressing and closed with foil flaps (Fig. 1). For air transport, the patient was secured onto a spinal board while being immobilized in a vacuum mattress.

**Fig. 2:**
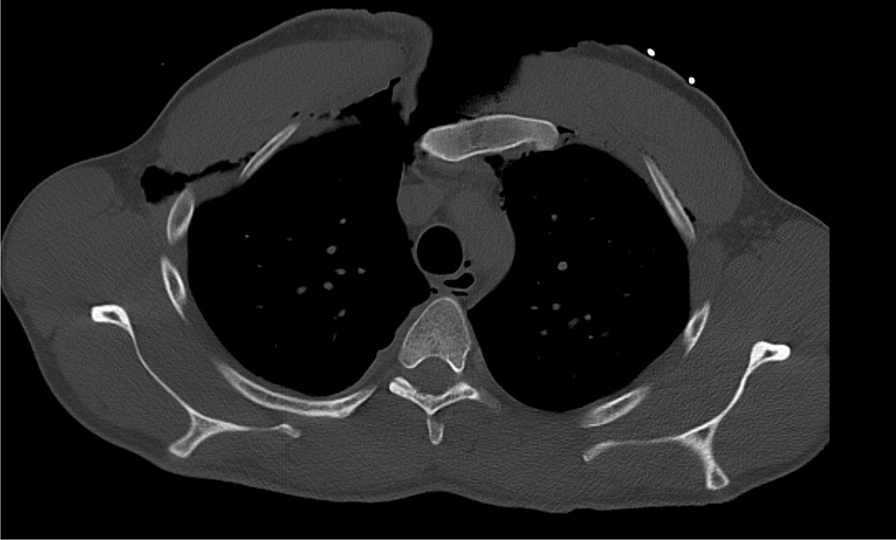
Axial view of the open thorax trauma with massive soft tissue damage on the proximal, right sternum

**Fig. 3:**
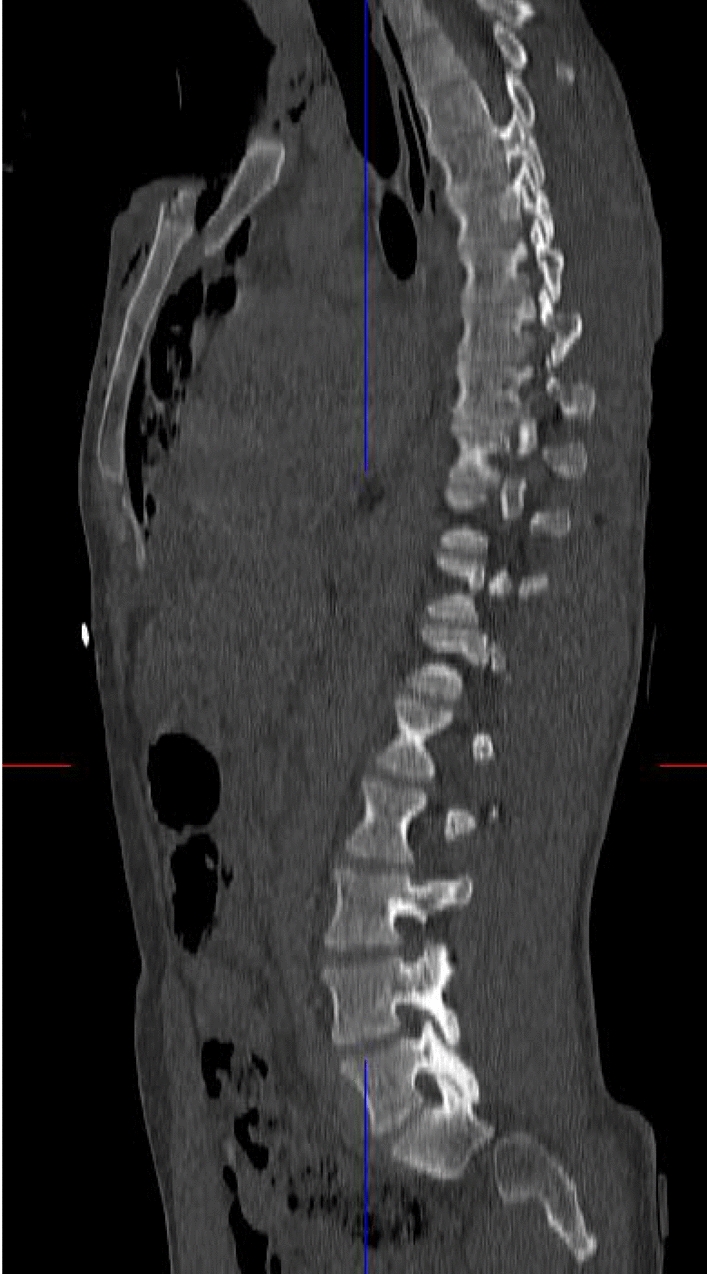
Sagittal plane of the CT showing the proximal fragment of the manubrium sterni being dislocated posteriorly. No injuries of the spine were detected

**Fig. 4:**
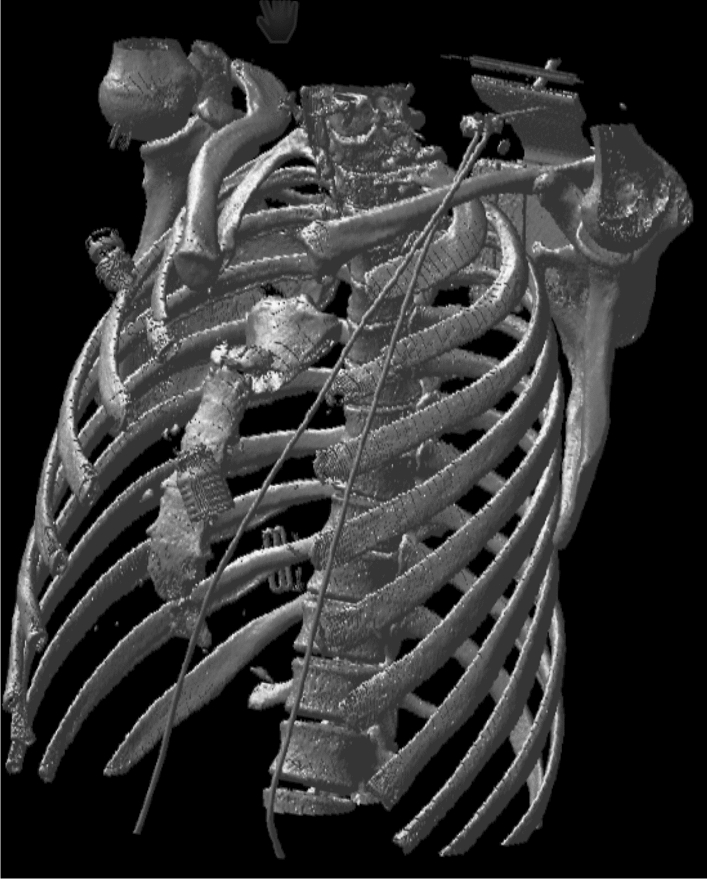
Three-dimensional reconstruction of the sternum fracture, bilateral SC joint dislocation. Furthermore, we can see bilateral dislocation of the first and second rib, with the second rib on the right having suffered a fracture in the ventral aspect

On arrival at the emergency department the patient was haemodynamically stable. Ventilation was continued without complications despite the pleural laceration. The patient received an antibiotic single shot with 1.5 g intravenous cefuroxime (Zinacef^®^, Hikma Pharmaceuticals) due to the open fracture (Tscherne and Oestern Grade III) [6]. A trauma assessment following Advanced Trauma Life Support (ATLS^®^) guidelines was performed, which revealed no additional injuries. The additionally performed focused assessment with extended focused assessment with sonography in trauma (eFAST) revealed no free fluid in the abdominal cavity or rectovesical excavation. The thoracic laceration was roughly inspected and re-covered with sterile gauze and closed with foil. The patient, who remained hemodynamically stable, then underwent a polytrauma whole-body computed tomography (CT) scan (Figs. 2, 3, 4). The scan showed a transverse fracture of the manubrium sterni with posterior displacement of the manubrium and anterior dislocation of both clavicles in the SC joints with no evidence of a fracture of the clavicles. Besides a right sided hemopneumothorax there were bilateral dislocations of the first and second ribs at the sternocostal joints. The second rib on the right side was fractured in an oblique manner (type B2) and the second rib on the left side was fractured in a complex, noncomminuted way (C1) [7].

After initial diagnostics and treatment in the emergency department, the patient was transferred to the operating room to receive immediate multidisciplinary surgery.

### Operating room

A thoracic surgeon began the procedure with wound exploration and debridement. The thorax cavity was open with exposed pericardium and exposed visceral pleura. The right mammary artery had to be ligated due to a traumatic transection. The pleura parietalis was sutured bilaterally using a single-button technique with 3–0 monofilament absorbable sutures (Polydioxane PDS, Ethicon Inc., Johnson and Johnson, Raritan, New Jersey, USA), and bilateral chest tubes (24Ch) and a retrosternal drainage tube (24Ch) were inserted into the mediastinum after extensive lavage of the mediastinum and thoracic cavities.

**Fig. 5:**
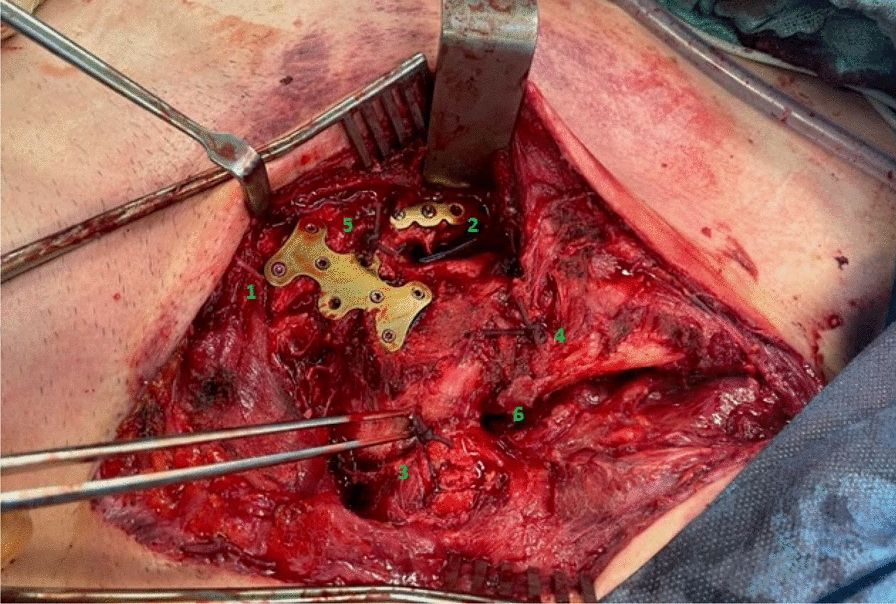
Intraoperative image after surgical stabilization of the open sternal fracture and the dislocated SC joints. 1: Inserted plate osteosynthesis of the sternum. 2. Plate osteosynthesis of the second rib on the right. 3: Refixed left SC joint. 4: Refixed right SC joint. 5: Refixed right costosternal joint. 6: Open mediastinum

Osteosynthesis of the open sternal fracture was then performed. A locking compression plate (LCP) 2.4/2.7 with variable angle and six holes from the forefoot/midfoot system by Synthes (Synthes Inc., West Chester, Pennsylvania, USA) was used to reduce and fix the dorsally displaced sternum fracture in an off-label use manner. The appropriate screws were inserted at a stable angle. A six-hole Matrix Rib universal plate was used for osteosynthesis of the second right rib. Again, the appropriate screws were inserted at a stable angle. The dislocated sternoclavicular and costosternal joints were repositioned with a trans osseous cerclage using a 1.5 mm polydioxane (PDS) cord. Further stabilization and compression were achieved by looping the PDS cord in a figure of eight through the appropriately drilled holes. The intraoperative situs is shown in Fig. 5 with visible plates and refixed SC joints.

After successful osteosynthesis, primary wound closure was then performed. Complete reconstruction of the pectoralis muscle was not possible due to a large defect in the muscle fibres.

### Postoperative treatment

**Fig. 6:**
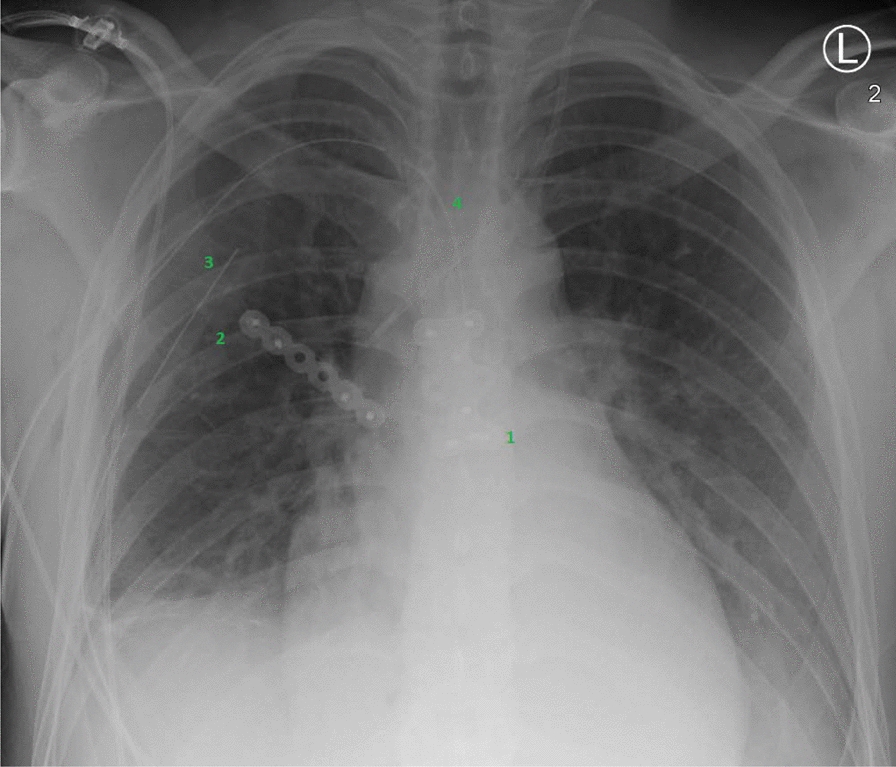
Postoperative chest overview. 1: Inserted plate osteosynthesis of the sternum. 2: Inserted plate osteosynthesis of the second rib on the right. 3: Inset Bülau drainage on the right. 4: Inset drainage in the mediastinum. The inlaid Bülau drain on he left has already been removed

After surgical therapy, there were no cardiopulmonary complications during the intensive care unit stay and the patient was able to be extubated and transferred to an intermediate care unit the following day. Here the patient was monitored for 3 consecutive days, after which he was transferred to the general ward. During hospital stay, the patient received intravenous antibiotic therapy with cefuroxim 1.5 g 1–1–1 intravenously (Zinacef^®^, Hikma Pharmaceuticals) and 4.5 g 1–1–1–1 i.v. piperacillin/tazobactam (Tazobac^®^, Pfizer Inc.) for 11 days. Wound healing progressed well and showed no signs of infection. The postoperative x-rays are shown in Fig. 6. The patient was discharged without complications after a total of 12 days without further antibiotic therapy.

**Fig. 7:**
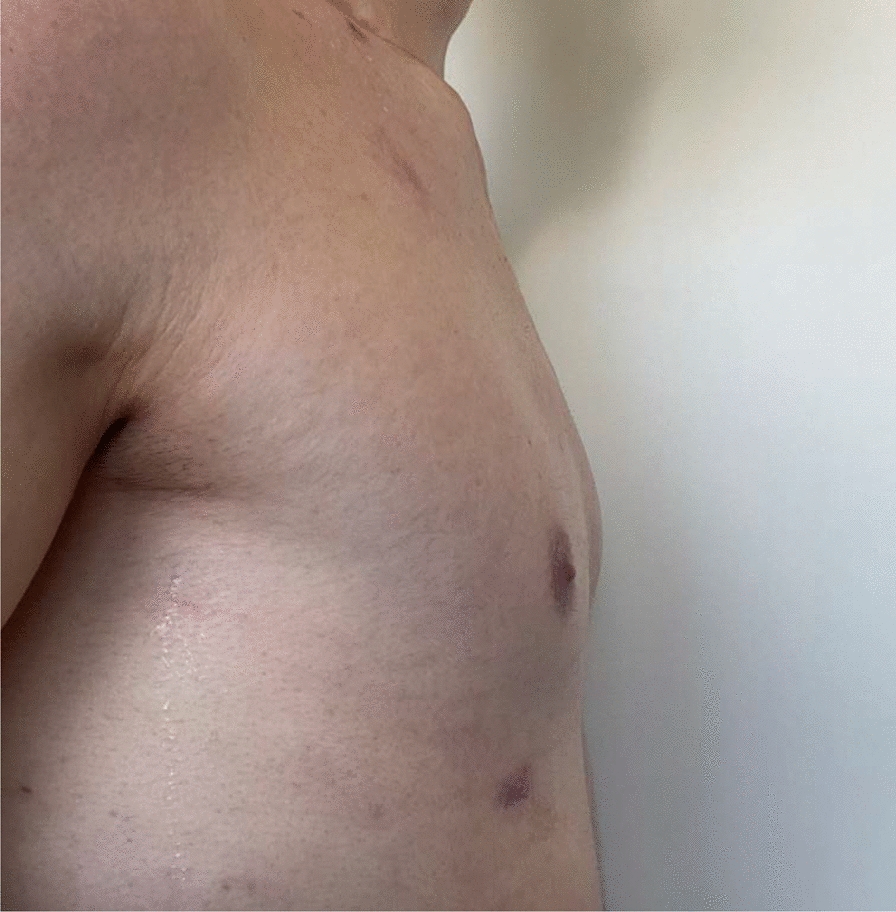
Photo documentation of the wound in a lateral view 6 weeks after surgical treatment

**Fig. 8:**
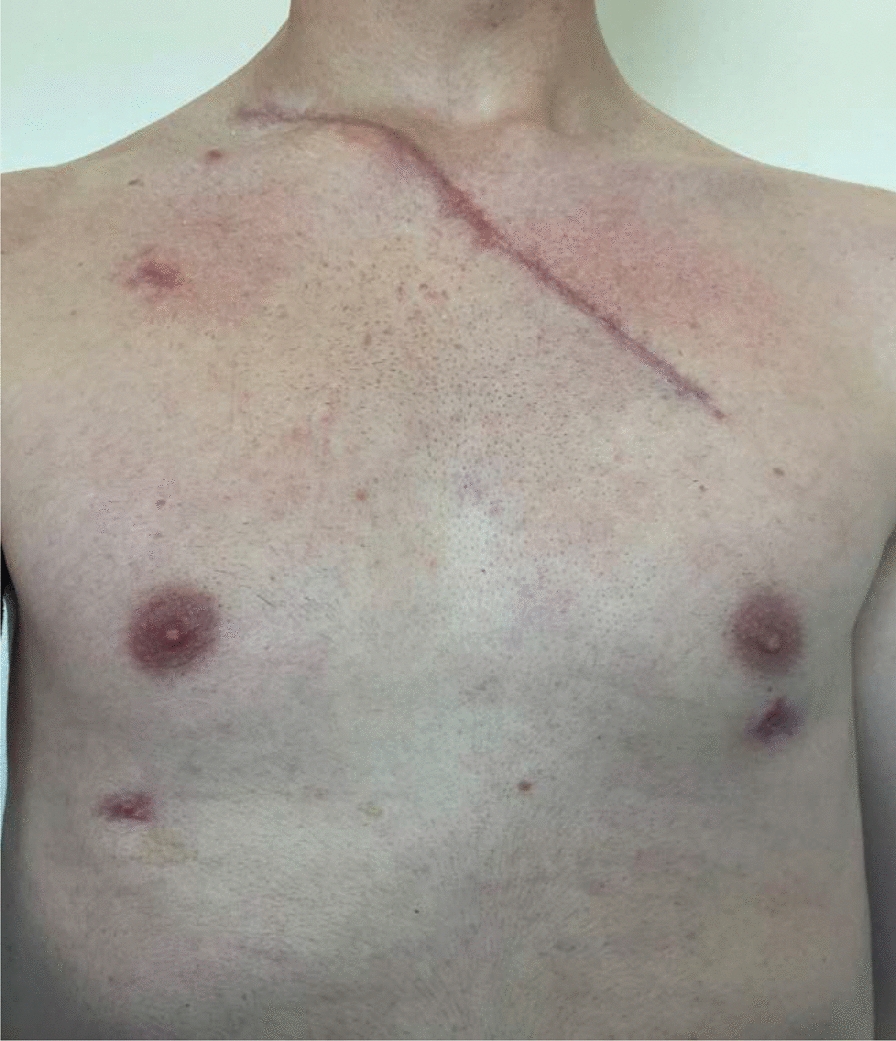
Photo documentation of the wound in a frontal view six weeks after surgical treatment

**Fig. 9:**
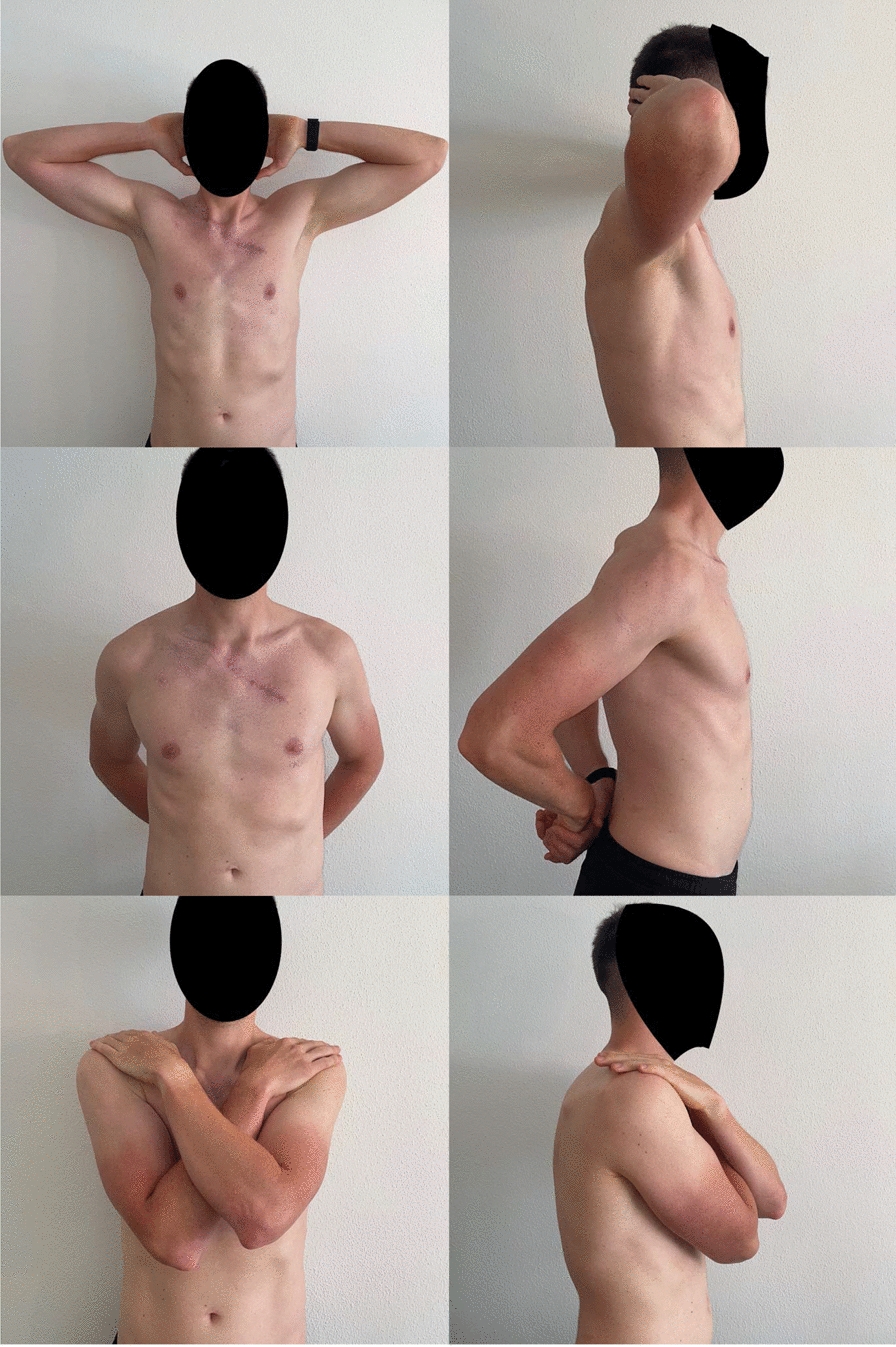
Clinical images of shoulder function 1 year postoperatively

At the 6-week follow-up, the wounds were completely healed as shown in Figs. 7, 8. The patient was able to lift both arms above his head without pain. During this follow-up, informed consent was obtained by the patient to retrospectively work up the case report and review of the literature. The patient underwent physical therapy three times per week. Now, 1 year after the treatment, he is back cycling on road and is playing tennis without limitations. There has been no recurrence of the SC joint dislocation, and the patient is not describing any symptoms of SC joint instability. In Fig. 9 the functionality of the shoulder and clavicle is demonstrated one year after surgical treatment.

Review and discussion

All nine cases of bilateral sternoclavicular joint dislocation that have been reported in the literature are shown in Table 1. From all the cases that have been described, five were of traumatic origin. These included one case of sternum fracture, one case of bilateral clavicle fracture, one case of bilateral epiphyseal fracture, and one case of multiple rib fractures. In our case, we found a combination of open thoracic trauma, rib, and sternum fracture next to the bilateral SC joint dislocation, which made surgical treatment challenging. Due to the open thoracic trauma, we opted for a single shot of 1.5 g of cefuroxim (Zinacef^®^, Hikma Pharmaceuticals) as is standard practice and recommended in international guidelines [8, 9]. It is worth noting that only one of the traumatic cases reported no concomitant injuries. This suggests that a high trauma mechanism is needed to cause this rare injury pattern, as was the case in our patient. The five cases were treated either surgically or conservatively. Surgical treatment included plate fixation of the sternum with subsequent reduction of the SC joints, joint capsule reconstruction, and temporary transarticular Kirschner wire fixation, as well as open reduction and internal fixation. In this case, we utilized a technique similar to the one used by Yi *et al.* by plating the sternum to achieve a more anatomically correct angle and joint positioning of the manubrium sterni and the SC joints [5]. Furthermore, we reconstructed the ligamentous capsule of the joints using PDS cord (Ethicon Inc., Johnson & Johnson, Raritan, New Jersey, USA) to enhance stability, comparable to the technique reported by Fandridis *et al.* [6].

**Table 1:** All literature in which bilateral SC joints are described

Source/Author	Mechanism	Concomitant injuries	Dislocation direction	Diagnostics	Treatment
Yi *et al.* [5]	Traumatic	Sternum fracture	Anterior	Rx + CT + 3D Reco	Surgical
Wang *et al.* [13]	Traumatic	Bilateral claviclefracture	Posterior	CT + 3D Reco	Surgical
Baumann *et al.* [14]	Traumatic	Bilaterale epiphyal fracture	Posterior	CT	Surgical
Albarrag *et al.* [10]	Traumatic	Blunt chest trauma	Anterior and posterior	CT + 3D Reco	Conservative
Gleason *et al.* [15]	Spontaneous	–	Anterior subluxation	CT	Conservative
Chien *et al.* [11]	Traumatic	Right femur fracture, multiple rib fractures	Anterior	Rx + CT + 3D Reco	Conservative
Nichols *et al.* [16]	Idiopathic	–	Anterior	CT Angio	Conservative
Echlin *et al.* [17]	Idiopathic	–	Anterior subluxation	CT + 3D	Conservative
Ege *et al.* [18]	Idiopathic	Brachiocephalic vein compression	Posterior	CT	Surgical SC Joint resection

### Conservative treatment

Two of the reported traumatic cases of bilateral SC joint dislocation were treated conservatively. Albarrag *et al.* ultimately advised the patient about surgical treatment concerning the posterior SC joint dislocation, but the patient chose the conservative route [10]. This consisted of initial immobilization followed by physical therapy and nonsteroidal anti-inflammatory drugs. The exact regimes are not mentioned in the paper. The other traumatic SC joint dislocation that has been treated conservatively, reported by Chien *et al.*, also does not share an exact conservative treatment plan [11]. In this case report, the patient had to be intubated due to the deteriorating pulmonary status, as the patient developed tachypnea up to a frequency of 34 breaths/minute. In our case, the patient was sedated and intubated upon the accidents site due to the deteriorating neurological status and diminishing protective reflexes. SC joint instability is often treated in a conservative manner and shows varying results [12]. In cases where stability cannot be obtained conservatively, surgical treatment can be discussed with the patient. In our case, conservative treatment was not an option given the life-threatening injury that the patient had suffered.

## Conclusions

The case report highlights the rarity of bilateral sternoclavicular dislocation combined with open thorax trauma in a cyclist. Immediate interdisciplinary intervention, including surgical repair and meticulous postoperative care, facilitated successful patient recovery. This underscores the critical role of comprehensive trauma management in complex polytrauma cases. In conclusion, this case report highlights the rarity and complexity of a traumatic injury involving bilateral sternoclavicular dislocation, with this case being the first case reported with concomitant open thorax trauma. Our patient benefited greatly from immediate air bound transportation to an interdisciplinary care provider which houses both thoracic and trauma surgery departments.

## Data Availability

Not applicable.

## Abbreviations

SC-joint: Sternoclavicular joint
ATLS: Advanced trauma life support
eFAST: Enhanced Focused Assessment with Sonography for Trauma
LCP: Locking compression plate
CT: Computed tomography
PDS: Polydioxane
